# Alleviation of Motor Impairments in Patients with Cerebral Palsy: Acute Effects of Whole-body Vibration on Stretch Reflex Response, Voluntary Muscle Activation and Mobility

**DOI:** 10.3389/fneur.2017.00416

**Published:** 2017-08-16

**Authors:** Anne Krause, Eckhard Schönau, Albert Gollhofer, Ibrahim Duran, Anja Ferrari-Malik, Kathrin Freyler, Ramona Ritzmann

**Affiliations:** ^1^Department of Sport Science, University of Freiburg, Freiburg, Germany; ^2^Center of Prevention and Rehabilitation, University of Cologne, Cologne, Germany

**Keywords:** spastic cerebral palsy, spasticity, supraspinal, spinal, voluntary movement control

## Abstract

**Introduction:**

Individuals suffering from cerebral palsy (CP) often have involuntary, reflex-evoked muscle activity resulting in spastic hyperreflexia. Whole-body vibration (WBV) has been demonstrated to reduce reflex activity in healthy subjects, but evidence in CP patients is still limited. Therefore, this study aimed to establish the acute neuromuscular and kinematic effects of WBV in subjects with spastic CP.

**Methods:**

44 children with spastic CP were tested on neuromuscular activation and kinematics before and immediately after a 1-min bout of WBV (16–25 Hz, 1.5–3 mm). Assessment included (1) recordings of stretch reflex (SR) activity of the triceps surae, (2) electromyography (EMG) measurements of maximal voluntary muscle activation of lower limb muscles, and (3) neuromuscular activation during active range of motion (aROM). We recorded EMG of m. soleus (SOL), m. gastrocnemius medialis (GM), m. tibialis anterior, m. vastus medialis, m. rectus femoris, and m. biceps femoris. Angular excursion was recorded by goniometry of the ankle and knee joint.

**Results:**

After WBV, (1) SOL SRs were decreased (*p* < 0.01) while (2) maximal voluntary activation (*p* < 0.05) and (3) angular excursion in the knee joint (*p* < 0.01) were significantly increased. No changes could be observed for GM SR amplitudes or ankle joint excursion. Neuromuscular coordination expressed by greater agonist–antagonist ratios during aROM was significantly enhanced (*p* < 0.05).

**Discussion:**

The findings point toward acute neuromuscular and kinematic effects following one bout of WBV. Protocols demonstrate that pathological reflex responses are reduced (spinal level), while the execution of voluntary movement (supraspinal level) is improved in regards to kinematic and neuromuscular control. This facilitation of muscle and joint control is probably due to a reduction of spasticity-associated spinal excitability in favor of giving access for greater supraspinal input during voluntary motor control.

## Introduction

For decades, whole-body vibration (WBV) has widely been applied in different areas of rehabilitative medicine, geriatrics, and as a training method for elite athletes (1). More recently, the respective research has increasingly been focused on the application and potential benefits of WBV as a therapy in neuro-rehabilitation, such as in adolescent patient groups with spastic cerebral palsy (CP) (2). With the advantage of great neuroplasticity in children, training regimes commonly achieve higher efficiency compared to adolescent patient groups (3, 4). To date, scientific evidence about possible beneficial effects of WBV on spasticity in CP has only reported the outcome, such as functional parameters of movement control. This is, for example, assessed by the clinical diagnostic tool “(Modified) Ashworth Scale” (5, 6). Apart from the fact that this scale has been characterized with significant limitations of reliability (7–9) and validity for children with CP (10), objective neurophysiological diagnostics, which provide a deeper understanding of WBV and its underlying mechanisms, are lacking.

Depending on the respective origin, severity and position of the lesion, spastic CP as a neurological motor disorder leads to several motor impairments (11, 12). Spasticity, as one of the most prominent spinal phenomena, is defined as a motor disorder with velocity-dependent elevated tonic stretch reflexes (SRs) and exaggerated tendon jerks (13). Those include hyperexcitability of short-latency (14, 15), but reduced long-latency reflexes (16). Coexisting with spasticity, also muscle weakness (17, 18) as well as diminished selective and voluntary supraspinal motor control (19) are expressed in developmental disorders of functional movement: patients suffer from gross motor problems (12), such as impaired mobility (20) and postural control (19, 21), as well as disturbances in gait (22–24). On a neuromuscular basis, reduced electromyographic activity (22), a strong co-activation of antagonistic muscles during movement (15, 22, 23, 25), diminished reciprocal inhibition (17, 26), and changes in perception (12) have been associated with those functional impairments and should be considered regarding therapy of movement disorders.

The benefits of WBV include its uniqueness as a *passive* training modality during which neuromuscular structures are modulated by mechanical oscillations of the support-surface (1, 27, 28). Thus, its application in neuro-rehabilitation has emerged as a particularly valuable tool. The positive effects of WBV exercise for CP involve acute (29, 30) and long-term training-induced adaptations (31–36). Those are well documented on a functional level: while acute modulations comprise enhancements in gait-related parameters (30, 37) and joint mobility, mutually accompanied with decreased spasticity (29), long-term adaptations also include improvements of strength (2, 32, 33), increased ankle excursions (34), gross motor functioning (2, 37), and balance (32). Nevertheless, those results predominantly focus on the effects of WBV on a functional level, but evidence about the underlying mechanisms is still deficiently researched for patients with neurological disorders. Based on evidence that WBV has an impact on the central motor control in a healthy population, such as a decrease of spinal excitability and Ia afferent reflex activation after WBV (38–41), it could have an impact on deficiently controlled neuromuscular structures in subjects with CP. Nonetheless, a transfer of investigations in a neuro-rehabilitation setting with objective assessment tools is still lacking.

For this purpose, the aim of the current study was to identify neurophysiological changes after WBV in children with spastic CP. In particular, it should be examined whether the phenomenon of reduced spinal excitability after WBV might lead to a reduction of spasticity-related parameters in neuromuscular control. Based on the deficient voluntary movement control in subjects with CP, our approach included three protocols with electrophysiological and functional assessments that are of relevance during everyday movements: besides (1) controlling SR activity, changes were examined including (2) voluntary neuromuscular activation and (3) active joint mobility in the lower limb.

We hypothesized that WBV reduces SR responses, and thus reduces exaggerated sensory input via Ia afferent pathways. We further expected that this release in the spasticity-related condition would produce a better movement control reflected by a reduction in co-contraction and enhanced joint mobility during voluntary activation.

## Materials and Methods

### Subjects

Over 9 months (June 2014–February 2015) subjects with spastic CP were medically examined by the attending physicians in terms of the inclusion criteria of the study, such as diagnosis of unilateral or bilateral spastic CP [gross motor function classification system (GMFCS) score 2–4], the ability to stand upright with support, and healthy cognitive performance, so that procedures were understood by the subjects. Exclusion criteria were neurosurgical procedure of nerve structures (rhizotomy) (29), acute injuries, and anxiety. For the screening process, subjects underwent physical and medical examination so that 44 subjects could be identified to fit the abovementioned criteria (18 females, 26 males, age 8 ± 4 years, height 123.0 ± 20.2 cm, body mass 26.3 ± 14.1 kg, GMFCS score 2.6 ± 0.5). Based on reliability and task performance ability, subject numbers varied between protocols. The exact numbers are noted below for each single protocol. All subjects and parents gave written informed consent to the diagnostic procedure in accordance with the latest revision of the Declaration of Helsinki, which is approved by the ethics committee of the University Hospital of Cologne.

### Procedures

**Figure 1:**
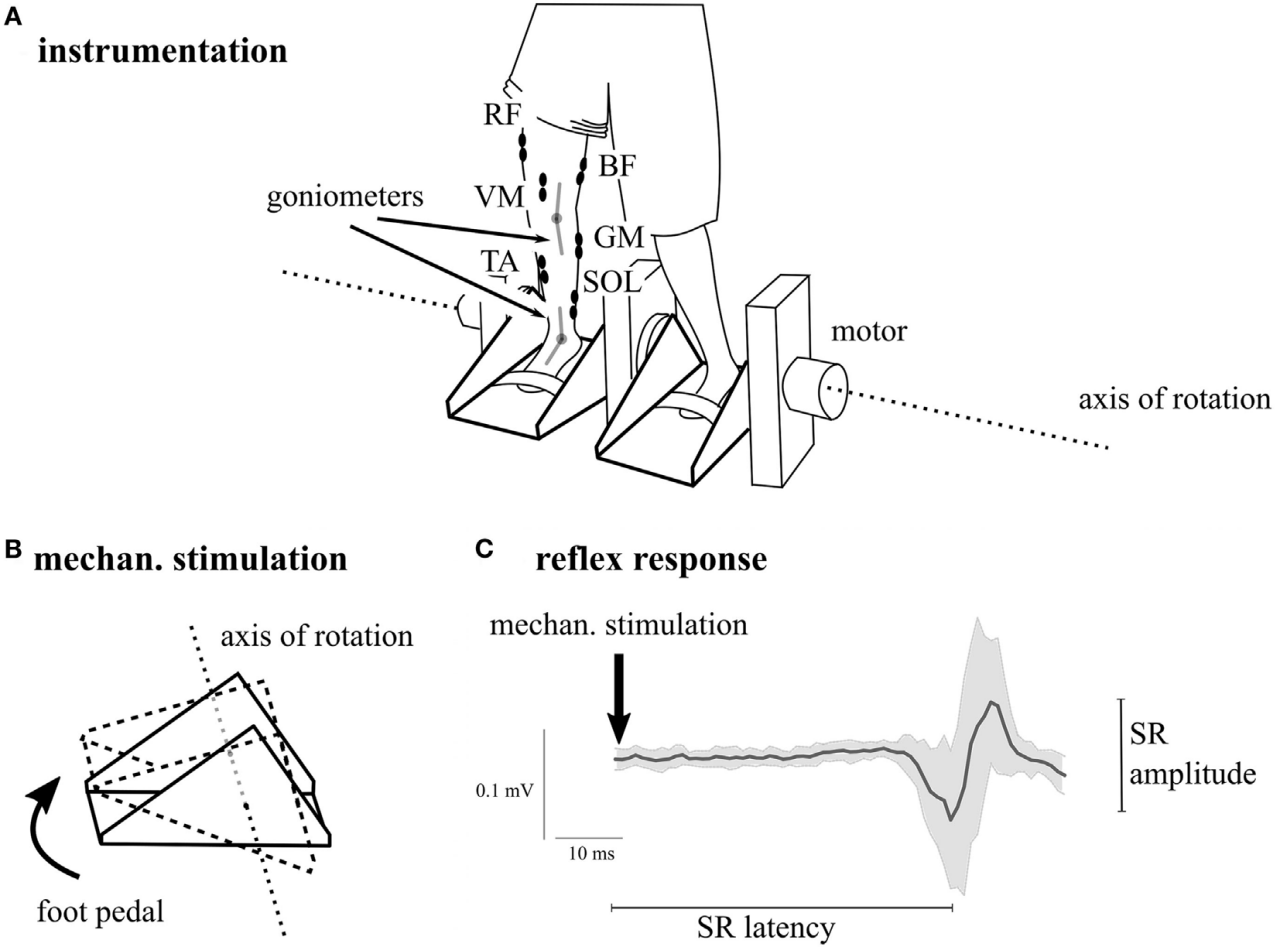
Illustration of the overall subject preparation and the instrumentation in *Protocol 1*. The participant was prepared with surface electromyography of the lower limb muscles m. soleus (SOL), m. gastrocnemius medialis (GM), m. tibialis anterior (TA), m. rectus femoris (RF), m. vastus medialis (VM), and m. biceps femoris (BF) as well as electro-goniometry of the ankle and the knee joint. In *Protocol 1*, stretch reflexes (SRs) were elicited mechanically by a dorsiflexion induced with the ankle ergometer **(A,B)**. Muscular responses **(C)** were recorded continuously; SDs are illustrated in gray.

We used a single-group repeated-measures study design to evaluate acute effects of WBV on neuro-mechanical coupling and motor control within a population of patients suffering from CP. For that purpose, kinematic and neuromuscular activation during motor tasks were recorded prior and immediately after 1-min bout of WBV (42, 43) in three different protocols: (1) the first protocol aimed to assess vibration-induced effects on the mechanically evoked SR in m. triceps surae (Figure 1). In the second protocol, (2) the influence of WBV on the maximal voluntary muscle activation of shank and thigh muscles was investigated. The third protocol (3) quantified the influence of WBV on the active range of motion (aROM) in the ankle and knee joint. Thus, vibration was applied for each protocol and between protocols a short break was included (>10 min) to minimize fatigue. Measurements were documented, surveyed, and supervised by the authors of the study. Prior to the first measurement, tasks and machinery were demonstrated to the subjects and practiced to exclude the influence of habituation effects within results and to avoid subjects’ anxiety of the investigation’s procedure.

Outcome measures were recorded twice prior to WBV under the same conditions with short breaks (>10 min) to ensure that changes were not based on habituation or learning effects and to exclude subjects from the data analysis who were unable to repeat the tasks reliably in the required paradigms. Subjects who demonstrated great variations (>40%) between two separated pre-measurements were excluded. In detail, maximal 12 subjects (of 37, *Protocol 1*), 11 subjects (of 23, *Protocol 2*) and 16 subjects (of 27, *Protocol 3*) had to be excluded. All other subjects were included to calculate mean values (as described in “Calculation of Outcome Parameters”).

### Whole-body Vibration

We used a side-alternating vibration platform (Galileo Sport, Novotec Medical, Pforzheim, Germany), which generates vibration by platform oscillations along the frontal plane (28). Subjects were placed on the upper surface of the platform, centrally aligned over the axis of rotation, with their feet parallel about 8.5–17.0 cm apart from the axis of rotation, depending on the respective body height (1). The given distances resulted in a range of vibration amplitude of 1.5–3 mm (peak-to-peak displacement 3–6 mm). Independently from vibration amplitude, the frequency was set individually for each subject, ranging between 16 and 25 Hz, based on the subjects’ abilities (peak acceleration ranging between 15 and 48 m s^−2^). Exposure to WBV was set for 1 min (42, 43), with subjects maintaining a static body position with a knee angle of 10°, forefoot stance and equal weight distribution on both feet (44). Neuromuscular and kinematic assessments were conducted immediately following WBV exposure.

### Protocols

For all three protocols, subjects were prepared for kinematic and neuromuscular assessment: first, monoaxial electro-goniometers (Biometrics^®^, Gwent, UK) were attached to lower limb joints, with the rotation axis being placed on the malleolus lateralis of the ankle and over the lateral knee joint center, respectively. The moveable arms of the goniometers were lined up with the longitudinal axis of the foot and shank (ankle) as well as with the longitudinal axis of the shank and femur (knee) (44). In addition, an EMG of the three shank muscles m. soleus (SOL), m. gastrocnemius medialis (GM), and m. tibialis anterior (TA), and the three thigh muscles m. biceps femoris (BF), m. rectus femoris (RF), and m. vastus medialis (VM) was used to assess muscular activation. Therefore, bipolar Ag/AgCl surface electrodes (Ambu Blue Sensor P and N, Ballerup, Denmark; diameter 7 and 9 mm, center-to-center distance 25 mm) were attached onto the disinfected skin over the belly of the respective muscle according to surface electromyography (EMG) for the non-invasive assessment of muscles (45). A reference electrode was placed over the tibia. Cables were connected to the electrodes and fixed to the skin. Signals were transferred to an amplifier, where data were band-pass filtered (10 Hz–1.3 kHz) and amplified (1,000×).

All signals were recorded synchronously with a sampling frequency of 1,000 Hz.

#### *Protocol 1*—SRs

In the first protocol, SR responses in the m. triceps surae were evoked in 37 subjects by a motor-driven ankle ergometer (Figure 1), dorsiflexing the foot passively (40). SRs were recorded with SOL and GM EMG, while mechanical dorsiflexion was monitored with goniometry. Visually, TA EMG was monitored as well to ensure that TA was not stimulated simultaneously. The axis of the ankle joint coincided with the rotation axis of the ankle ergometer. The mechanically induced dorsiflexion at the ankle joint were applied with an amplitude of 8.7 ± 0.4° and a velocity of 108.7 ± 5.2°/s, evoking an SR with interindividual latencies ranging between 35 and 55 ms (Figure 1; Table 1). Subjects stood upright in the ergometer holding onto sidebars for body stabilization and extending their knees as much as possible considering the respective degree of spasticity. Standardized position was monitored with the assessment of background EMG in the shank muscles as well as ankle and knee joint position prior to the stimulus. Instructions were given and controlled as follows: standing still, looking straight ahead, and not counteracting the passive ankle rotation actively. In total, 2 × 15 stimulations with interstimulus intervals of 4 s were applied prior and after WBV, respectively (40).

**Table 1:** Results of stretch reflex (SR, *Protocol 1*), maximal voluntary muscle activation (VA, *Protocol 2*), and active range of motion (aROM, *Protocol 3*) normalized to baseline values (=100%).

		Pre	Post	SD (±)	Percentage changes (%)	*p*-Value
*Protocol 1*—SR	Latency m. soleus (SOL) SR	1.00	1.01	0.02	+1	**0.01**
	Amplitude SOL SR	1.00	0.88	0.16	−12	**<0.01**
	Latency m. gastrocnemius medialis (GM) SR	1.00	1.01	0.03	+1	**0.01**
	Amplitude GM SR	1.00	0.95	0.30	−5	0.20

*Protocol 2*—VA	SOL	1.00	1.19	0.52	+19	
	GM	1.00	1.37	1.03	+37	
	m. Tibialis anterior (TA)	1.00	1.15	0.69	+15	**0.03**
	m. Rectus femoris (RF)	1.00	1.10	0.61	+10	
	m. Biceps femoris (BF)	1.00	1.16	0.61	+16	
	m. Vastus medialis (VM)	1.00	1.03	0.43	+3	

	Quotient SOL VA/SR	1.00	1.19	0.41	+19	**0.04**
	Quotient GM VA/SR	1.00	0.92	0.71	−8	0.32

*Protocol 3* —aROM	Ankle joint excursion	1.00	0.99	0.42	−1	0.46
	Knee joint excursion	1.00	1.15	0.20	+15	**<0.01**

	SOL/TA	1.00	1.54	1.45	+54	
	GM/TA	1.00	1.92	2.01	+92	
	TA/SOL	1.00	2.17	2.72	+117	
	TA/GM	1.00	1.40	1.21	+40	**0.01**
	BF/RF	1.00	1.76	2.17	+76	
	BF/VM	1.00	1.67	2.32	+67	
	RF/BF	1.00	1.35	1.73	+35	
	VM/BF	1.00	1.63	2.26	+63	

*Bold numbers demonstrate significant time effects from pre- to post-measurements with p < 0.05*.

#### *Protocol 2*—Maximal Voluntary Muscle Activation

In 23 subjects, maximal voluntary muscle activation (VA) was assessed by EMG with movement instructions according to Daniels and Worthingham (46). Subjects were seated in an upright body position with the head facing in a forwards position; hands were positioned next to the body. For 5 s, muscular activation was performed isometrically against manual resistance given by the operator. Instructions depended on the respective muscle recorded: shank muscles were measured with the knee joint extended, extending (plantarflexion—SOL and GM) or flexing the ankle joint (dorsiflexion—TA) as much as possible against manual resistance. To quantify RF and VM activity, from a flexed starting position of 90° in the knee joint, a knee joint extension was performed by the subject against manual resistance. Finally, for BF measurements, subjects were instructed to flex their knee against manual resistance given to the calf (46, 47). Fatigue of the muscles was prevented by including recovery pauses between measurements.

#### *Protocol 3*—aROM

In the same seated position as described in *Protocol 2*, 27 subjects were asked to actively bend and extend their ankle and knee joint as much as possible (29). In the end position of angular excursion, the maximal voluntary rotation around joint axes (aROM) was measured by goniometry. In addition, muscular activation was recorded with EMG to analyze the amount of activation of the agonist in comparison to the respective amount of activation of the antagonist (co-activation ratio) during aROM. Ankle joint excursions, implemented by plantarflexion and dorsiflexion, were therefore recorded in combination with activity of shank muscles, while knee joint excursion was combined with muscle assessment of the BF, RF, and VM (48, 49).

### Data Processing

*Protocol 1*: Peak-to-peak amplitudes and integrals of SOL SR and GM SR were averaged over 30 trials, each recorded before and after WBV. Characteristics of the SR were assessed between the initial deflection of the EMG signal from baseline to the second crossing of the baseline according to Petersen et al. (50). SOL SR and GM SR latencies (ms) were determined visually by the time interval between stimulus artifact and the first slope of the averaged SR amplitude, according to Ritzmann et al. (51).

As muscle pre-activity and the joint position could affect the SR beyond the WBV treatment, both items were strictly controlled for all participants. Muscular pre-activation was controlled by an assessment of the rectified and integrated background EMG [iEMG (mVs)] for the interval of 100 ms prior to dorsiflexion (52). Ankle and knee joint deflections were controlled by the goniometric recordings (°) for the intervals of 20 ms prior to dorsiflexion (52).

*Protocol 2*: VA of the skeletal muscles was assessed according to Kellis and Katis (53). Values were rectified and integrated [iEMG (mVs)].

**Figure 2:**
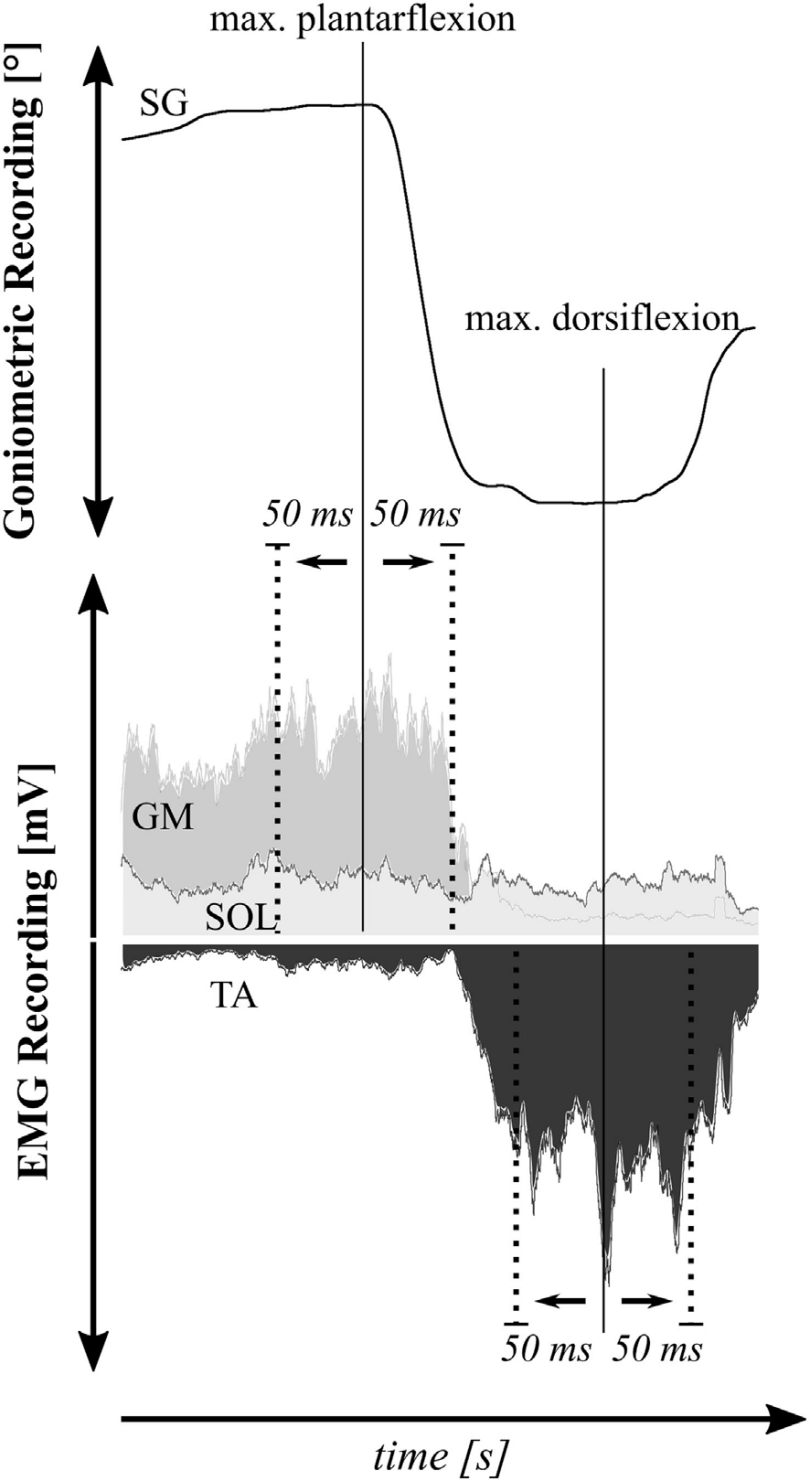
Exemplary illustration of the co-activation ratio determination in the shank: EMG activity of agonists was evaluated in relation to antagonists 50 ms before and 50 ms after peak joint excursion during max. plantarflexion for m. soleus (SOL) EMG and m. gastrocnemius medialis (GM) EMG and during maximal dorsiflexion for m. tibialis anterior (TA) EMG.

*Protocol 3*: Ankle and knee joint displacements during aROM were assessed based on the maximal amplitudes of goniometric recordings (°). Voluntary activation of the agonistic and antagonistic muscles involved in the flexion and extension of the ankle (TA, SOL, and GM) and knee joint (BF, RF, and VM) was analyzed within 50 ms before and 50 ms after peak joint excursion (Figure 2). EMG values were rectified, integrated, and expressed as iEMGs (mVs).

### Calculation of Outcome Parameters

All values are expressed as mean values and SDs for the conditions before and after WBV. For pre-data, mean values were calculated from both pre-measurements. Data regarding voluntary muscle activation (VA—*Protocol 2*; co-activation ratio—*Protocol 3*) and joint excursions (*Protocol 3*) were normalized to baseline values obtained before WBV and outcome parameters were expressed as percentage changes.

In addition, to quantify the relation between voluntary and reflex activity, VA integrals were divided by the corresponding SR integral (VA/SR-ratio). This ratio illustrates the direct ratio between voluntary (with input from a supraspinal level) and reflex-associated motor control (of spinal origin). To gain further insight into antagonistic muscle coordination, the co-activation ratio of antagonistic muscles was evaluated for the end position of the aROM obtained in *Protocol 3* (Figure 2): therefore, the iEMGs of the agonists in the lower limb were divided by their respective iEMGs of antagonists (SOL/TA, GM/TA, BF/RF, and BF/VM) according to the calculation of Duchateau and Baudry (54). The higher the percentage changes from pre- to post-WBV, the better the muscle coordination and the smaller the passive and active counterforces produced by antagonists.

### Statistics

To test for time effects in *Protocol 1*, paired Student’s *t*-tests were performed with the variable time (2, pre vs. post) concerning the SR amplitudes and latencies. For *Protocol 2 and 3*, time effects were assessed by one factor repeated measures analysis of variance applied for aROM (goniometry) and muscular activation (VA, co-activation ratio). Within-subject factors with variables time (2, pre vs. post) and muscle activity (6, SOL vs. GM vs. TA vs. RF vs. BF vs. VM) (VA, *Protocol 2*) or muscle groups (8, agonists vs. antagonists) (co-activation ratio, *Protocol 3*) were defined. Greenhouse–Geisser correction was used in case of violation of the assumption of sphericity (tested with Mauchly’s test for sphericity). *p* < 0.05 was defined as the significance level. To determine changes between pre- and post-measurements of VA/SR-ratio, one-tailed paired Student’s *t*-tests with *p* < 0.05 were calculated.

Test–retest reliability estimates were computed by the intraclass correlation coefficient for *Protocols 2 and 3* using a one-way random single measure model with two items as both pre-measurement time points. Outcomes were described by Cronbach’s α according to Fleiss (55). For absolute indices, standard error of measurement (SEM) was calculated according to Harvill (56).

Statistics were conducted and analyzed by using the software IBM SPSS Statistics 22 (SPSS, Inc., Chicago, IL, USA). Data are presented in mean values and SDs (mean ± SD) with post-values being normalized to baseline values.

## Results

Grand means and SDs are displayed in Table 1.

**Figure 3:**
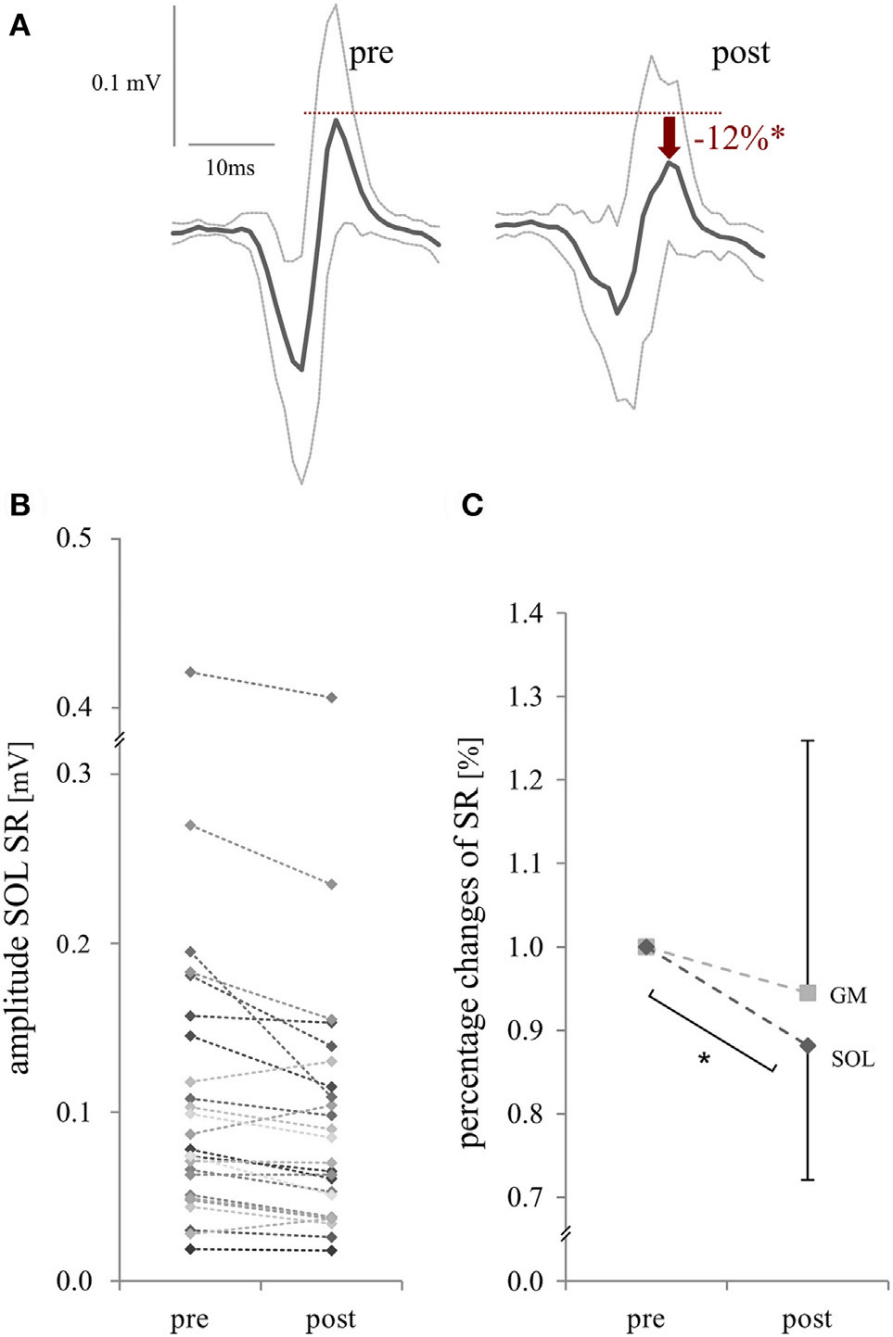
Changes in stretch reflex (SR) excitability in the m. triceps surae, induced by mechanical dorsiflexion of the ankle joint (*Protocol 1*): representative SR amplitude [m. soleus (SOL)] of a single subject **(A)**, results of each subject [SOL, **(B)**] and mean values for SR excitability [SOL and m. gastrocnemius medialis (GM), **(C)**] before (pre) and after whole-body vibration (post). **(A,B)** Raw values of reflex amplitude and **(C)** percentage changes for the m. triceps surae are presented. Significant changes (*p* < 0.05) are illustrated with a * symbol.

*Protocol 1*: Mechanically evoked SOL SR were significantly reduced after acute exposure to WBV (−12 ± 16%, *p* < 0.01), changes observed for GM SR remained not significant (−5 ± 30%, *p* = 0.20, Figure 3). Latencies increased in SOL SR and GM SR by +1% (*p* = 0.01) corresponding to 0.5–1.0 ms, respectively. Muscular pre-activation prior to stretch and kinematic starting position (goniometry) in the ankle and knee joint remained unchanged with respect to different points in time (cf. Table 2, A). Reliability was assured with good to excellent Cronbach’s α at levels of 0.88–0.96 for muscular pre-activation and 0.91–0.99 for initial joint angles, respectively.

**Table 2:** Absolute [standard error of measurement (SEM)] and relative test–retest reliability (Cronbach’s α) was evaluated with the initial position from pre- to post-WBV (*Protocol 1*, A) and between both pre-measurements (*Protocol 2 and 3*, B).

A		Pre	Post	Cronbach’s α	SEM
*Protocol 1*	m. Soleus (SOL) pre-activation (mVs)	5.23^−2^ ± 1.41^−3^	5.25^−2^ ± 1.47^−3^	0.884	5.00^−4^
	m. Gastrocnemius medialis (GM) pre-activation (mVs)	5.46^−2^ ± 1.59^−3^	5.47^−2^ ± 1.55^−3^	0.957	3.22^−4^
	Ankle joint position (°)	32.2 ± 20.6	31.1 ± 20.3	0.991	1.9
	Knee joint position (°)	24.2 ± 20.4	21.6 ± 20.2	0.914	5.9

**B**		**Pre 1**	**Pre 2**	**Cronbach’s α**	**SEM**

*Protocol 2*	SOL (mVs)	0.011 ± 0.005	0.012 ± 0.006	0.914	0.002
	GM (mVs)	0.018 ± 0.012	0.021 ± 0.017	0.935	0.004
	m. Tibialis anterior (TA) (mVs)	0.084 ± 0.082	0.076 ± 0.071	0.990	0.007
	m. Rectus femoris (RF) (mVs)	0.059 ± 0.031	0.068 ± 0.040	0.903	0.012
	m. Biceps femoris (BF) (mVs)	0.052 ± 0.053	0.056 ± 0.063	0.985	0.008
	m. Vastus medialis (VM) (mVs)	0.078 ± 0.068	0.095 ± 0.090	0.972	0.015
*Protocol 3*	Joint excursion ankle (°)	28.3 ± 7.9	27.5 ± 8.5	0.943	2.9
	Joint excursion knee (°)	42.6 ± 17.3	45.9 ± 21.1	0.960	4.0
	SOL/TA	0.49 ± 0.42	0.42 ± 0.21	0.600	0.13
	GM/TA	0.47 ± 0.37	0.67 ± 0.42	0.884	0.14
	TA/SOL	11.10 ± 8.12	7.91 ± 6.79	0.931	1.78
	TA/GM	16.05 ± 13.52	12.00 ± 10.85	0.939	2.68
	BF/RF	2.96 ± 2.55	2.53 ± 2.03	0.737	1.04
	BF/VM	7.74 ± 10.62	8.14 ± 15.08	0.955	3.20
	RF/BF	2.95 ± 3.27	2.64 ± 3.08	–	–
	VM/BF	1.57 ± 1.09	2.12 ± 3.02	0.380	2.37

*Absolute values are illustrated as means ± SD*.

**Figure 4:**
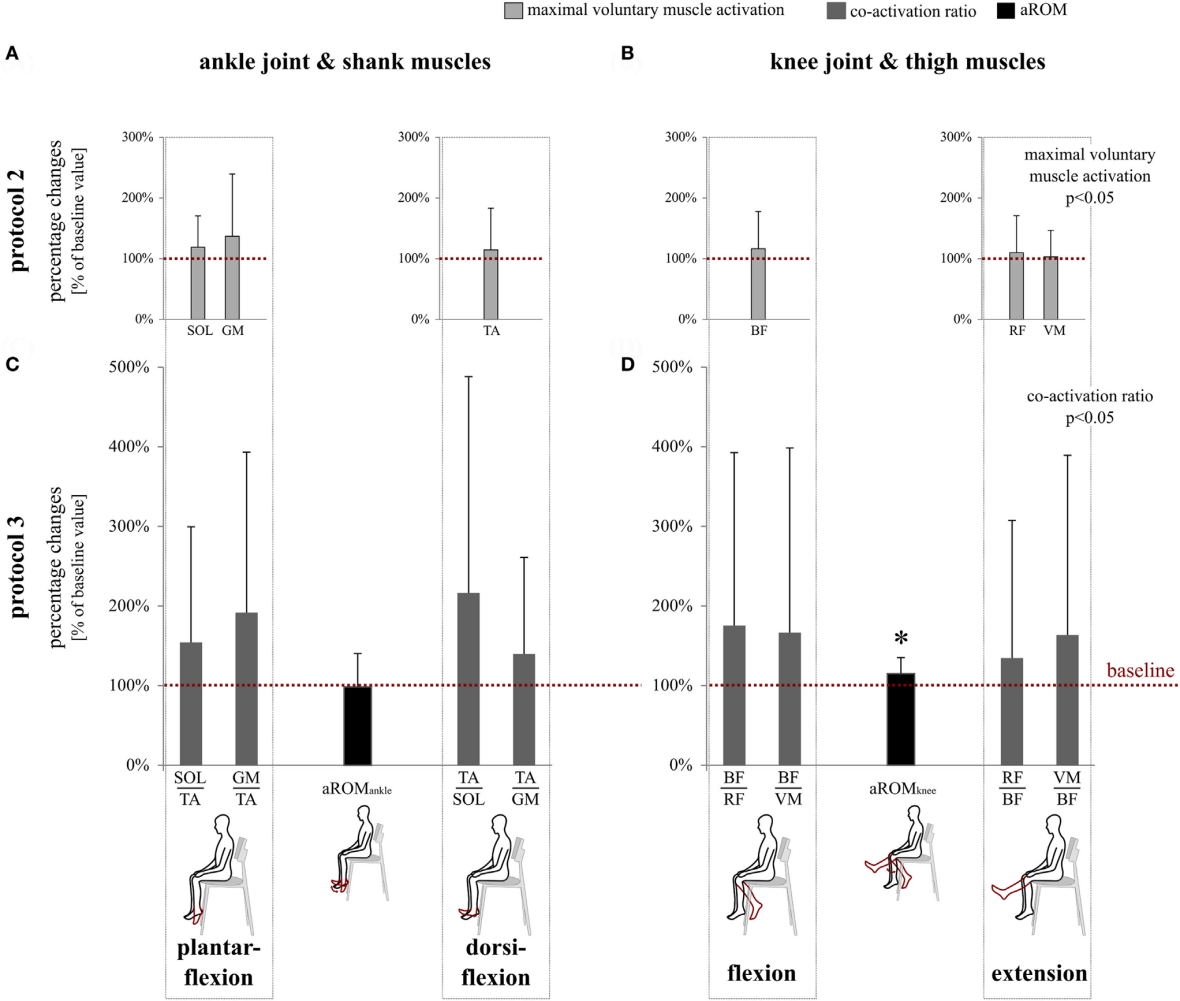
This graph illustrates percent changes in neuromuscular and kinematic parameters. The red dotted lines serve as references and mark baseline values at 100% referring to data collected before whole-body vibration (WBV). **(A)** Modulations of maximal voluntary muscle activation for the antagonist muscles encompassing the ankle joint [m. soleus (SOL), m. gastrocnemius medialis (GM), and m. tibialis anterior (TA)] and **(B)** knee joint [m. biceps femoris (BF), m. rectus femoris (RF), and m. vastus medialis (VM)] for joint flexion and extension. The rmANOVA revealed a significant increase (*p* < 0.05) in response to WBV. **(C)** WBV-induced modulations in co-activation ratios of the muscles surrounding the ankle joint and **(D)** knee joint and corresponding active ranges of motion (aROM). The rmANOVA revealed a significant increase in co-activation ratios (*p* < 0.05) and knee joint excursion (*p* < 0.05) in response to WBV. Data are normalized to control values obtained prior to WBV. Significant changes in aROM are illustrated by *p < 0.05.

*Protocol 2*: Significant pre- to post-effects were observed after WBV: iEMGs during VA were significantly elevated for all lower limb muscles (*p* = 0.03). No interaction effects of time x muscle were observed (*p* = 0.78). While VA/SR-ratio in SOL was distinctly increased (19 ± 41%, *p* = 0.04), no changes could be observed for GM (−8 ± 71%, *p* = 0.32) (Figure 4). Cronbach’s α yielded excellent results of 0.90–0.99. SEM values are listed in Table 2 (B).

*Protocol 3*: No significant changes could be observed in angular excursion of the ankle joint (−1 ± 42%, *p* = 0.46). In the knee joint, however, active angular excursion was increased during flexion and extension (+15 ± 20%, *p* < 0.01), accompanied by significantly greater co-activation ratios over time reflected by values >1 for all recorded agonist–antagonist muscle pairs (*p* = 0.01). No interaction effects were revealed for the variables time × muscle group (*p* = 0.86). Cronbach’s α was excellent for kinematic (0.94–0.96) and acceptable for neuromuscular measures (0.73–0.96). Test–retest reliability was questionable for co-activation during plantarflexion (SOL/TA) and unacceptable for knee extension (RF/BF and VM/BF, cf. Table 2, B). SEM values are listed in Table 2 (B).

## Discussion

The current study demonstrated acute modulations after a 1-min bout of WBV including (1) decreased SOL SR activation, (2) elevated maximal voluntary muscle activation in lower limb muscles, and (3) increased aROM in the knee joint only, accompanied by improved intermuscular coordination in subjects with CP. The results point toward modulations opposed to the characteristics of pathological movement disorders and indicate improved voluntary movement control.

*Voluntary and Involuntary Movement Control*: Changes of the muscle and its neuronal control are causal for impairments in CP children (17, 57), including spasticity-associated hyperexcitability of spinal reflexes (58, 59). In the current study, neurophysiological consequences following WBV were investigated in regard to spinal excitability: subjects displayed a reduction of reflex activity by −5 to −12%, with minimally prolonged latencies of 1% in the m. triceps surae—the muscle that is closest to the vibration platform, and thus most affected by the WBV stimulus (44). These results are in line with those observed in healthy subjects (38–40) and demonstrate in general that modulations in SR sensitivity to WBV may be comparable to subjects suffering from CP. Vibration is known to affect the receptor organs and reduce the sensitivity of primary or secondary muscle spindle endings (60–63). Furthermore, the integration of muscle spindle input is changed as well, which is illustrated by changes of Ia afferent transmission. Spasticity is, among others, associated with a decrease of inhibition of reflex activity (64), and thus by lowering the Ia afferent sensory input, spinal excitability might become comparable to that existing in healthy subjects (40). A reduction of SR sensitivity is of functional relevance during simple movement, because the muscle does not overreact to small stretch loads, leading to better “access” for voluntary muscle activation. This is in accordance with results demonstrating corticospinal facilitation (65) concomitant with spinal inhibition (66) after local vibration applied in healthy subjects. Therefore, facilitated input from supraspinal and brain-descending (corticospinal) structures could be associated with enhanced movement control (67, 68).

In spasticity, reciprocal inhibition is pathologically affected (64, 69–71). This can be reflected in pathological co-contractions of antagonistic muscles leading to augmented joint stiffness (20, 22) as well as muscle weakness leading to deficient movement patterns in children with CP (17, 72). In the current study, however, neuromuscular control during voluntary movement execution emphasizes improved neuromuscular coordination following WBV: the results clearly demonstrate an enhanced muscular activation of lower limb muscles (values up to +37%) as well as improved coordination between agonists and antagonists during maximal excursion of the ankle and knee joint (ratios up to +117%). Especially, modulations in the m. triceps surae are of great relevance, as this muscle is predominantly affected in children with CP (69). It could point toward a possible involvement of reciprocal inhibition as being facilitated after WBV, which would be in line with evidence concerning modulations during local vibration (73), involving a neuronal inhibitory modulation of the non-vibrated muscle (74).

*Functional Relevance and Application*: From a functional point of view, current neuromuscular modulations clearly point toward improved voluntary motor control which is assumed to be guided by supraspinal structures after WBV. For instance, a reduction of reflex activity has previously been associated with greater functional performance such as postural control (75–77) or concerning walking ability in subjects with CP (78). In accordance with the neuromuscular benefits, greater motor control is also displayed by a wider range of motion in the knee joint, which is in line with previous investigations (29).On the basis of this interrelation between spasticity, joint angular velocity and the ability for gross motor functioning performance (79), increased joint mobility contributes to a better functional performance in subjects suffering from CP. Another aspect deals with the reflex latencies, which are decreased in subjects suffering from spasticity (80). WBV-induced minimally prolonged reflex latencies indicating a reduced transmission velocity over afferent and efferent nerve fibers, which may counteract the pathological symptoms in CP patients (80). With an emphasis on leg muscles which are of great relevance for everyday life activities due to their involvement in locomotion and posture control (2, 36, 37), these modulations provide a perspective for advantageous WBV training regimes applied in the field of neuro-rehabilitation.

*Limitations*: The main difficulty of the current investigation was to balance standardization and moral support to the young participants while securing ethical tenability during the assessments. As a result, effects were controlled by test–rest reliability, but not with a separate control group. For instance, in *Protocol 3*, neuromuscular activation failed reliability regarding knee joint excursion, and thus results should be considered with caution. The demonstrated effects after WBV emphasize the beneficial application of vibration. However, without any comparison with other treatments, it cannot be concluded whether those effects are solely specific to vibration. In addition, even though variables (type, amplitude, and frequency) of the vibration protocol were chosen carefully, based on current evidence, immediate results following WBV cannot be generalized and information about the temporal maintenance of the current modulations needs to be addressed in future research. Therefore, the evaluation of vibratory effects on spasticity during the described protocols can solely be discussed on the basis of evidence described in the literature.

### Conclusion and Prospective

In conclusion, the current study demonstrates an acute modulation of motor control in spastic CP children after WBV. Reduced pathological reflex responses concomitant with an increased voluntary muscular activation, improved intermuscular coordination of antagonists and increased knee joint mobility might be interpreted as counteracting spasticity-associated deficits in children with CP. With the benefit of a passive training modality, it may be assumed that spasticity and weakness in CP patients may be acutely and favorably modulated by the vibration stimulus. In addition, as the current young subject group probably still underlies a reorganization and maturation process of the developing brain, this could be particularly beneficial for achieving neural adaptation, and thus preventing secondary structural changes. Based on the positive acute effects in children with CP, investigations should be accelerated to illustrate the effect of longer periods of vibration and long-term adaptations in this patient group. Nevertheless, by demonstrating improved voluntary movement execution after WBV, the time frame immediately after WBV may be used for targeted movement therapy: subjects might actually take advantage of increased supraspinal input by means of greater voluntary motor control which has to be investigated in future studies.

## Ethics Statement

The current study is in accordance with the latest revision of the Declaration of Helsinki, which is approved by the ethics committee of the University Hospital of Cologne. All subjects and parents gave written informed consent to the diagnostic procedure in accordance with the latest revision of the Declaration of Helsinki, which is approved by the ethics committee of the University Hospital of Cologne. Children with spastic cerebral palsy were involved in the current study. They were medically examined by the attending physicians regarding inclusion criteria of the study and both, children and parents, were informed about the procedure. Children and parents gave their consent to take part but could drop out at any time during the measurements without stating any reason.

## Author Contributions

All the authors AK, ES, AG, ID, AF-M, KF, and RR made substantial contributions to the conception or design of the work, the acquisition, analysis, and interpretation of data for the work. Further, they contributed drafting the work and revising it critically, they helped with the final approval of the version to be published, and made the agreement to be accountable for all aspects of the work in ensuring that questions related to the accuracy or integrity of any part of the work are appropriately investigated and resolved.

## Conflict of Interest Statement

The authors declare that the research was conducted in the absence of any commercial or financial relationships that could be construed as a potential conflict of interest.

## Abbreviations

aROM, active range of motion; BF, musculus biceps femoris; CP, cerebral palsy; EMG, electromyography; GM, musculus gastrocnemius medialis; GMFCS, gross motor function classification system; ICC, intraclass correlation coefficient; iEMG, rectified and integrated EMG; m., musculus; RF, musculus rectus femoris; rmANOVA, repeated measures analysis of variance; SENIAM, surface electromyography for the non-invasive assessment of muscles; SOL, musculus soleus; SR, stretch reflex; TA, musculus tibialis anterior; VA, maximal voluntary muscle activation; VA/SR-ratio, maximal voluntary muscle activation divided by stretch reflex; VM, musculus vastus medialis; WBV, whole-body vibration.
